# Terabase-Scale Coassembly of a Tropical Soil Microbiome

**DOI:** 10.1128/spectrum.00200-23

**Published:** 2023-06-13

**Authors:** Robert Riley, Robert M. Bowers, Antonio Pedro Camargo, Ashley Campbell, Rob Egan, Emiley A. Eloe-Fadrosh, Brian Foster, Steven Hofmeyr, Marcel Huntemann, Matthew Kellom, Jeffrey A. Kimbrel, Leonid Oliker, Katherine Yelick, Jennifer Pett-Ridge, Asaf Salamov, Neha J. Varghese, Alicia Clum

**Affiliations:** a Joint Genome Institute, Lawrence Berkeley National Laboratory, Berkeley California, USA; b Physical and Life Sciences Directorate, Lawrence Livermore National Laboratory, Livermore, California, USA; c Applied Math and Computational Research Division, Lawrence Berkeley National Laboratory, Berkeley, California, USA; d Department of Electrical Engineering and Computer Sciences, University of California, Berkeley, California, USA; e Life & Environmental Sciences Department, University of California Merced, Merced, California, USA; University of Mississippi

**Keywords:** metagenomics, terabase, tropical soil, redox, rare biosphere

## Abstract

Petabases of environmental metagenomic data are publicly available, presenting an opportunity to characterize complex environments and discover novel lineages of life. Metagenome coassembly, in which many metagenomic samples from an environment are simultaneously analyzed to infer the underlying genomes’ sequences, is an essential tool for achieving this goal. We applied MetaHipMer2, a distributed metagenome assembler that runs on supercomputing clusters, to coassemble 3.4 terabases (Tbp) of metagenome data from a tropical soil in the Luquillo Experimental Forest (LEF), Puerto Rico. The resulting coassembly yielded 39 high-quality (>90% complete, <5% contaminated, with predicted 23S, 16S, and 5S rRNA genes and ≥18 tRNAs) metagenome-assembled genomes (MAGs), including two from the candidate phylum *Eremiobacterota*. Another 268 medium-quality (≥50% complete, <10% contaminated) MAGs were extracted, including the candidate phyla *Dependentiae*, *Dormibacterota*, and *Methylomirabilota*. In total, 307 medium- or higher-quality MAGs were assigned to 23 phyla, compared to 294 MAGs assigned to nine phyla in the same samples individually assembled. The low-quality (<50% complete, <10% contaminated) MAGs from the coassembly revealed a 49% complete rare biosphere microbe from the candidate phylum FCPU426 among other low-abundance microbes, an 81% complete fungal genome from the phylum Ascomycota, and 30 partial eukaryotic MAGs with ≥10% completeness, possibly representing protist lineages. A total of 22,254 viruses, many of them low abundance, were identified. Estimation of metagenome coverage and diversity indicates that we may have characterized ≥87.5% of the sequence diversity in this humid tropical soil and indicates the value of future terabase-scale sequencing and coassembly of complex environments.

**IMPORTANCE** Petabases of reads are being produced by environmental metagenome sequencing. An essential step in analyzing these data is metagenome assembly, the computational reconstruction of genome sequences from microbial communities. “Coassembly” of metagenomic sequence data, in which multiple samples are assembled together, enables more complete detection of microbial genomes in an environment than “multiassembly,” in which samples are assembled individually. To demonstrate the potential for coassembling terabases of metagenome data to drive biological discovery, we applied MetaHipMer2, a distributed metagenome assembler that runs on supercomputing clusters, to coassemble 3.4 Tbp of reads from a humid tropical soil environment. The resulting coassembly, its functional annotation, and analysis are presented here. The coassembly yielded more, and phylogenetically more diverse, microbial, eukaryotic, and viral genomes than the multiassembly of the same data. Our resource may facilitate the discovery of novel microbial biology in tropical soils and demonstrates the value of terabase-scale metagenome sequencing.

## INTRODUCTION

Metagenomics projects are producing terabases (Tbp) of sequence data (1–3), with some 2.5 petabases (Pbp) of metagenome data currently in the NCBI SRA database. These data sets are an opportunity to better understand environments and biogeochemical processes, to probe the rare biosphere (low-abundance microbes [4–6]), and to discover new lineages of life (7). However, using the existing wealth of metagenomic data sets to achieve these goals is difficult because the computational task of reconstructing an environment’s underlying microbes from sequencing reads, known as genome assembly (8), can be prohibitively computationally expensive for large data sets (9). Because of this limitation, the data analysis workflows available to most researchers rely on assembling metagenome samples one at a time, a process called multiassembly, followed by the complicated process of removing redundancy resulting from multiple samples of the same environment. Multiassembly is commonly used because the de Bruijn graph-based assembly software’s (10) memory requirements increase with sequence complexity and are exceeded at the terabase scale on single compute nodes. Resource limitations on sequencing capacity may present researchers with the choice of sequencing more samples at less depth, or fewer samples at more depth. These tradeoffs may result in a shallower accounting of microbial communities’ full complexity, with incomplete, fragmented metagenome-assembled genomes (MAGs); a bias toward the most abundant community members; and the absence of rare biosphere MAGs.

Coassembling (simultaneous assembly of multiple samples) metagenome samples from the same environment, possibly at the terabase scale, is a promising approach (11), provided that the petabytes of memory potentially required by de Bruijn graph assemblers can be accommodated. To this end, we previously developed MetaHipMer (11), a metagenome assembler that runs distributed across hundreds or thousands of compute nodes to coassemble terabases of metagenomic data, with assembly quality comparable to the state-of-the-art assemblers metaSPAdes (12) and MEGAHIT (13). That study (11) provided a proof of principle, illustrating that metagenome coassembly yields more contiguous assemblies representing more of an environment’s underlying genome sequences, with less redundancy, and no requirement for complicated deduplication procedures, than multiassembly (while multiassembly remains useful due to, in addition to its computational feasibility, better characterization of abundant genomes with high strain variation).

In this paper, we present the coassembly, along with the resulting MAGs and functional annotations, of 3.4 Tbp of metagenome sequence data derived from soil in the Luquillo Experimental Forest (LEF) in Puerto Rico, a Long Term Ecological Research Network site (14). Our goal is to demonstrate the potential wealth of biological discoveries that can be driven by large-scale metagenomic sequencing and coassembly and to provide a resource of MAGs and associated functional annotation for an environment in which terabases of sequence are available. The coassembled data came from the Great Redox Experiment (GRE) (15), a study focused on how redox oscillation frames the activities of microbial communities in humid tropical soils. The experiment used replicate soil incubations under oscillating oxic and anoxic conditions, along with ^13^C plant biomass amendments to perform stable isotope probing (SIP) experiments, resulting in 95 metagenome samples, which were sequenced at the DOE Joint Genome Institute (proposal: Microbial Carbon Transformations in Wet Tropical Soils: Effects of Redox Fluctuation https://doi.org/10.46936/10.25585/60000880). A coassembly of this scale of data was only possible using MetaHipMer on a supercomputing cluster, in this case the Oak Ridge National Laboratory’s Summit system (16). Metagenome binning of the coassembly contigs resulted in MAGs from a broad array of phyla, including some candidate groups, a substantial increase in the number of both MAGs and phyla over the individual metagenome samples’ assemblies (referred to collectively as the multiassembly). Low-abundance microbes and viruses absent in the multiassembly, and a mostly complete fungal genome and several unicellular eukaryotic MAGs, were found in the coassembly. The coassembly, MAGs, and annotations, along with search and analysis tools, are available in the IMG/MER Database (17).

## RESULTS AND DISCUSSION

### Metagenome coassembly.

A total of 3.4 terabase pairs (Tbp) of metagenome sequence data were coassembled to yield 75 Gbp of sequence in 55 million scaffolds (Table 1). Consistent with our previous finding (11) that coassembly results in improved contiguity, the coassembly scaffold *L*_50_ value is >3 times greater, and the number of scaffolds larger than 50 kbp is ~27-fold more, than in the multiassembly (combined individual metaSPAdes [12] assemblies). The coassembly thus has substantially more of the lengths of scaffolds interesting to biologists: those which may harbor genes, operons, and viruses and facilitate more accurate MAG extraction (18). Alignment of 22,666,691,390 reads to coassemble contigs of ≥500 bp resulted in 19,830,702,257 (87.5%) aligned reads (the median for individual assemblies of the GRE data was 51.2%). The length-weighted mean coverage of coassembly contigs was 40×. The alignment statistics indicate that substantially more of the LEF’s underlying microbial communities are being assembled in coasssembly than in multiassembly; nevertheless, 12.5% of the reads are not represented in the coassembly.

**TABLE 1:** GRE coassembly summary statistics^*a*^

Statistic	Coassembly	Multiassembly	Individual assembly (median)
No. of scaffolds	55,342,847	279,810,992	2,893,912
Scaffold (bp)	74,970,251,022	146,314,652,061	1,479,843,562
Scaffold *N*_50_	9,058,474	70,618,834	734,629
Scaffold *L*_50_ (bp)	1,656	526	517
Longest scaffold (bp)	1,464,928	1,406,223	80,051
No. of scaffolds >50 Kbp	26,818	1,000	3
Assembled sequence in scaffolds >50 Kbp (%)	3.25	0.05	0.015
Avg GC (%)	64.1	65.0	65.3

a Statistics from 95 individual assemblies (collectively referred to as the multiassembly) of the same data are shown for comparison.

We used metaSPAdes rather than MetaHipMer for the individual assemblies because, first, it is the standard Joint Genome Institute (JGI) metagenome assembler. Second, we could not use metaSPAdes for the coassembly, because a regression model based on the memory usage of previous metaSPAdes assemblies indicated that the 265 billion unique 31-mers in the GRE data would require 4.5 TB of memory to coassemble, 3-fold more than the capacity of the largest compute node we have access to. Coasssembling the GRE data with metaSPAdes would also require potentially several weeks of run time, whereas MetaHipMer completed the coassembly in about 1.5 h. We have previously extensively compared metaSPAdes and MetaHipMer and found them to be comparable in resulting assembly quality (11). Moreover, the independent Critical Assessment of Metagenome Interpretation 2 (CAMI2) contest also found MetaHipMer to be at least as good as metaSPAdes in quality (18).

### Annotation and MAG recovery.

The ~55 million assembled contigs were annotated and binned using the JGI metagenome workflow (19). In all, 112,151,078 genes were predicted, 99.4% of which are protein-coding genes (the remainder being RNA genes), with nearly all having some predicted function (Table 2). Metagenome binning resulted in 1,321 MAGs, 307 of which were high or medium quality (39 high and 268 medium) (20), and 1,014 of which were of low quality (which, because they are not made available in the IMG/MER Database, are provided in Table S1). The high- and medium-quality MAGs are made available on IMG and are the default set of MAGs discussed in this paper unless otherwise noted. These 307 MAGs represented 23 distinct phyla, including candidate groups *Dormibacterota* (candidate division AD3), *Eremiobacterota* (candidate division WPS-2), *Dependentiae* (candidate division TM6), and *Methylomirabilota* (candidate division NC10). With the exception of one medium-quality *Methylomirabilota* MAG in the multiassembly, these candidate phyla were absent from the 294 high- and medium-quality multiassembly MAGs, which in contrast, represented nine phyla (Table 3). Of note, the two *Eremiobacterota* (21) MAGs were of high quality (both >96% complete and <1% contaminated), indicating the potential of terabase-scale coassembly to produce reference-quality genomes for unculturable microbes.

**TABLE 2:** Summary statistics on the functional annotation of coassembly contigs

Type of gene	No.	Percent of total
All genes	112,151,078	
RNA genes	655,297	0.58
rRNA genes	26,420	0.02
5S rRNA	5,637	0.01
16S rRNA	8,150	0.01
18S rRNA	468	0.00
23S rRNA	11,438	0.01
28S rRNA	727	0.00
tRNA genes	628,877	0.56
Protein-coding genes	111,495,781	99.42
With product name	57,522,550	51.29
With COG	53,798,710	47.97
With Pfam	57,522,915	51.29
With TIGRfam	11,086,716	9.89
With SMART	11,999,050	10.70
With SUPERFam	55,768,333	49.73
With CATH FunFam	41,525,148	37.03
With KO	30,524,203	27.22
With enzyme	17,696,960	15.78
With MetaCyc	11,000,722	9.81
With KEGG	17,363,888	15.48
COG clusters	4,617	99.70
Pfam clusters	11,088	57.81
TIGRfam clusters	3,895	86.79
CRISPR count	31,334	

**TABLE 3:** Summary of coassembly MAGs, multiassembly MAGs, and species-level clusters (Mash distance of ≤0.05) per phylum^*a*^

Phylum	Coassembly MAGs (*n*)	Multiassembly MAGs (*n*)	Species-level clusters (*n*)
Acidobacteriota	59	71	64
Actinobacteriota	37	22	39
Bacteroidota	13	7	13
Bdellovibrionota	2		2
Chloroflexota	7		7
Cyanobacteria	1		1
Dependentiae	1		1
Desulfobacterota	1		1
Desulfobacterota_B	6		6
Dormibacterota	3		3
Eisenbacteria	2	25	4
Eremiobacterota	2		2
Fibrobacterota	1		1
Gemmatimonadota	1		1
Methylomirabilota	2	1	3
Myxococcota	29	15	32
Myxococcota_A	2	32	3
Patescibacteria	5		5
Planctomycetota	42		42
Proteobacteria	79	111	93
Spirochaetota	1		1
Verrucomicrobiota	7	10	8
Verrucomicrobiota_A	4		4
Total MAGs	307	294	-
Distinct phyla	23	9	-

a Notice that while the coassembly and multiassembly have similar total numbers of MAGs, the coassembly covers more than twice as many phyla and that species-level clustering of the coassembly and multiassembly MAGs combined reveals the considerable redundancy in the multiassembly (e.g., in the phyla Eisenbacteria and Myxococcota_A). Notice also that for a few phyla (e.g., Acidobacteriota and Proteobacteria) the multiassembly captures some species-level diversity that the coassembly does not. -, not applicable

**FIG 1:**
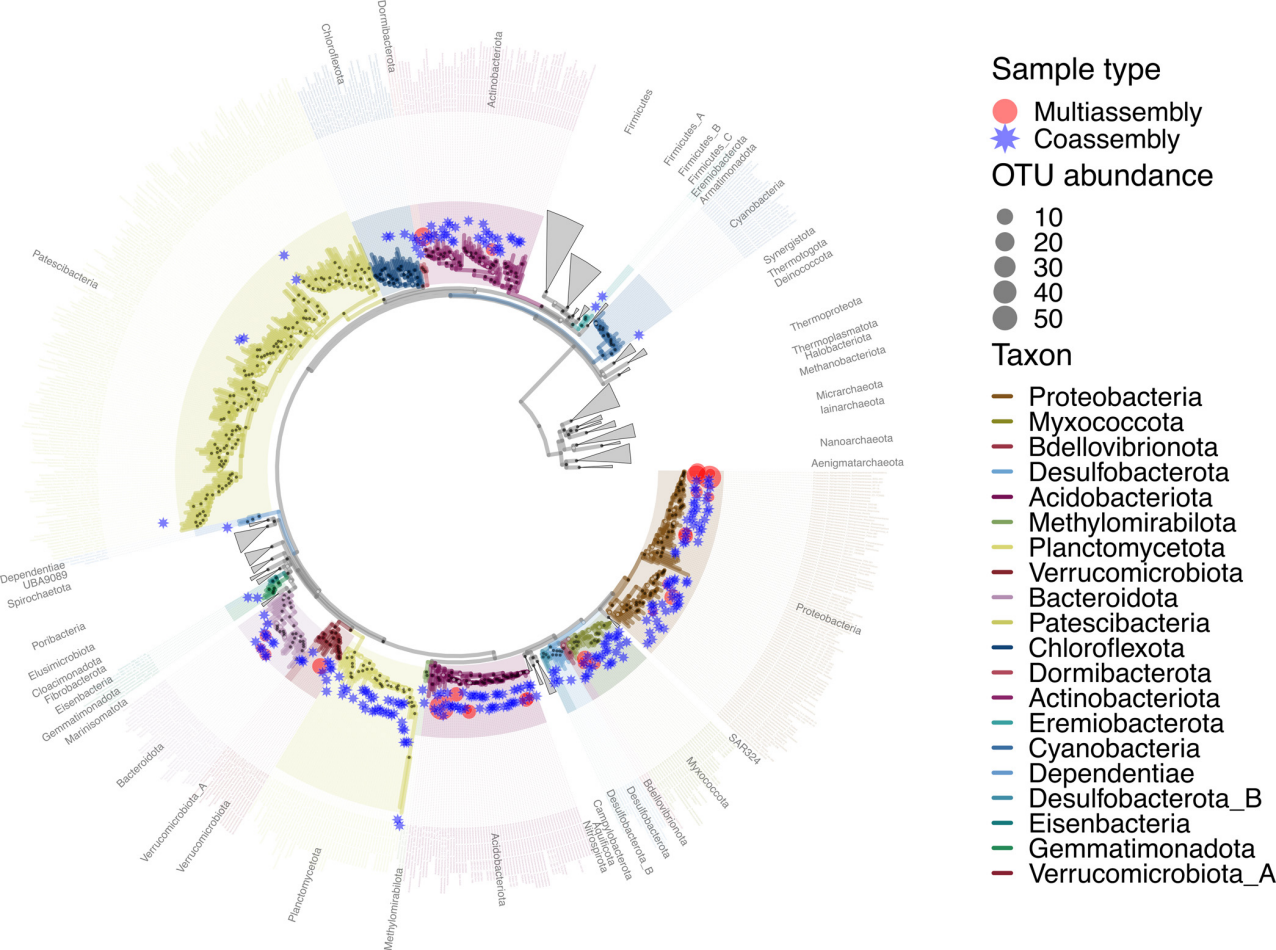
Phylogenetic overview of the MAG set (coassembly and multiassembly MAGs combined and dereplicated at 0.05 Mash pairwise distance, or approximately species level) from the Great Redox Experiment (GRE), a study of a humid tropical soil incubated under multiple redox conditions and amended with plant biomass. MAGs derived from either the multiassembly or coassembly are denoted by the red circles and blue stars, respectively. The phylogeny, consisting of MAGs and a reference set of genomes containing roughly genus- to family-level representatives spanning both bacterial and archaeal domains, was constructed by extracting, aligning, and concatenating the alignments of a set of three RNA polymerase subunit genes. Bubble sizes refer to the number of multiassembly MAGs within each Mash 0.05 cluster, noted by “OTU abundance” in the legend, and indicate community members assembled across multiple samples. All 307 coassembly MAGs were distinct species-level OTUs, whereas the 294 multiassembly MAGs contained 38 species-level OTUs. Phylum-level annotations are shown using GTDB-Tk taxon strings.

The RNA Pol-based phylogenetic tree in Fig. 1 provides an overview of the distribution of MAGs derived from our MetaHipMer coassembly versus the metaSPAdes multiassembly. Consistent with our finding of a broader array of phyla captured in the coassembly MAGs (Table 3), the phylogenetic diversity (PD) of coassembly MAGs was also far greater than that of the multiassembly MAGs (PD, 17.5 for the coassembly MAGs; PD, 3.4 for the multiassembly MAGs). Mash 0.05 clustering (22) of the 307 coassembly MAGs did not reveal any species-level redundancy, whereas clustering of the multiassembly MAGs reduced the species-level operational taxonomic unit (OTU) count to 38, indicating that the same community members were likely being assembled across samples (indicated by OTU abundance in Fig. 1). Of these 38 species-level OTUs from the multiassembly, 9 were found in the coassembly MAGs (using Mash 0.05 clustering), 298 clustered OTUs remained unique to the coassembly, and 29 OTUs were uniquely found in the multiassembly MAGs. The finding of some MAGs unique to the multiassembly is an expected result, as we previously found (11) that multiassembly better captures abundant, high-strain-variation genomes, while coassembly better captures low-abundance, rare genomes. At the phylum level, however, the coassembly MAGs contain more than twice as many taxa, and no taxa were uniquely found in the multiassembly MAGs.

### Rare biosphere.

We hypothesized that low-abundance rare biosphere microbes (4) might be present in the low-quality MAGs (completion, <50%; contamination, <10%) and might have low completeness due to low coverage in the data. Among the low-quality MAGs, we noticed a bin trivially lower than the medium-quality (MQ) completeness cutoff (49.1% completeness, 0.0% contamination) assigned to the candidate phylum FCPU426. FCPU426 has been detected in a 16S rRNA gene amplicon survey (23) and a function-driven single-cell genomics analysis (24) of different hot springs. Both studies estimated FCPU426’s abundance in its environment to be <1% of total microbial community composition. Here, the FCPU426 bin was covered at 23× in the coassembly. None of the 95 samples contributed more than ~1× coverage to the FCPU426 bin, indicating that this microbe would have been impossible to assemble using a multiassembly approach, as optimal microbial genome assembly typically requires >20× coverage (25). The NCBI taxonomy database currently has eight nucleotide sequences belonging to FCPU426, suggesting that, in addition to being low abundance in its environment, this candidate phylum is also poorly represented in genomic repositories.

Two low-quality bins with contamination slightly higher than the MQ cutoff were assigned to the genus *Labilithrix* (90.5% completeness, 10.32% contamination) and order UTPRO1 of the uncultured phylum *Binatota* (candidate division UBP10, 65.96% completeness, 10.53% contamination), covered at 19× and 55×, respectively. *Labilithrix* is a myxobacterium genus of the phylum *Myxococcota*, isolated from forest soil (26). *Labilithrix* is typically considered rare and it is of particular interest for secondary metabolite production capabilities along with other *Myxococcota* (27, 28). Myxobacteria grow as saprophytes on decaying organic matter, but under nutrient-limiting conditions, these bacteria form a cooperative multicellular swarm that becomes predatory (29). Given the supply of plant litter as a carbon source in these samples, it is reasonable to assume this *Labilithrix* organism was growing in its single cellular saprophyte low-density state; hence its low coverage. *Binatota*, on the other hand, is largely undescribed. *Binatota* genomes recovered from metagenome assemblies appear capable of aerobic methylotrophy, alkane degradation, and pigment production, but these have not yet been confirmed (30, 31). None of the 95 samples contributed more than 1× coverage to either of these bins, which again indicates the usefulness of coassembly for rare biosphere discovery.

In this data set, numerous LQ bins nearing the MQ completeness and contamination cutoffs have only phylum-level taxonomic assignment. For some of these unresolved bins, sequencing reads mapped from the 95 samples point to increased abundance specific to one of the redox treatment growth conditions relative to the others, including the carbon isotope supplied. Increased abundance under specific growth conditions could yield clues about the ecological niches these potentially novel taxa occupy that would have been missed with a multiassembly approach.

High- and medium-quality MAGs are often prioritized by biologists, but lower-quality MAGs, or even unbinned contigs, can enable insight into rare biosphere microbes. In the absence of targeted enrichment techniques (24), exhaustive sequencing may be required for sufficient coverage to resolve MAGs for the rarest microbes in an environment, which are often at <1% of total abundance. Our findings demonstrate the utility of massive metagenome sequencing and coassembly of the resulting data sets and suggest the possibility of coassembling agglomerated publicly available data sets (1, 3) for the discovery of rare biosphere microbes.

### Eukaryotic MAGs.

We hypothesized that other low-quality MAGs, or MAGs that do not even meet the MIMAG (20) standard for low quality due to high contamination, might be of eukaryotic origin. To test this hypothesis, we ran EukCC (32) to assign a eukaryotic origin to the low-quality MAGs. Applying the MIMAG (20) standards for medium- or higher-quality (completion, ≥50%; contamination, <10%), we identified one MAG at 81.2% completeness and 0.75% contamination, apparently belonging to the fungal class Sordariomycetes. The Sordariomycetes include the model fungus Neurospora crassa (33), important plant pathogens (34), and numerous endophytes (35). BUSCO (36) analysis, which estimates genome completeness based on the presence or absence of conserved single-copy orthologs, estimated the apparent fungal MAG to be 77.5% and 74.7% complete with respect to the fungal and Sordariomycetes lineages, respectively, roughly consistent with the EukCC completeness estimate. BLAST searches against reference databases indicated that this fungus is likely in the *Coniochaeta* clade (37), which includes fungal species found in decaying wood, leaf litter, and soil and likely represents a fungus occupying a saprotrophic role in the LEF soils. The fungal MAG’s coverage was 13× across all 95 samples, with no individual sample contributing more than 4× coverage; thus, it is unlikely this fungus could have been assembled using a multiassembly approach.

At lower completeness levels (10 to 50%), 30 additional partial eukaryotic MAGs were found, including multiple MAGs from the protist orders Tintinnida (5 MAGs), Diplonemea (5 MAGs), Diplomonadida (4 MAGs), and Hymenostomatida (2 MAGs). These may represent starting points for more detailed analyses of unicellular eukaryotes in LEF soils.

### Viruses.

To detect viral genomes in the GRE coassembly, assembled contigs were processed with a novel bioinformatic pipeline, geNomad (38). A total of 22,254 viral sequences were identified, of which 239 (1.1%) were found to represent high-quality genomes (estimated completeness, ≥90%) and 470 (2.1%) corresponded to medium-quality genomes (estimated completeness, 50 to 90%). Cross-referencing the identified viruses with medium- and high-quality MAGs revealed 44 proviruses integrated into 35 different genomes, mostly assigned to *Proteobacteria* (17 MAGs), *Planctomycetota* (8 MAGs), and *Acidobacteriota* (4 MAGs). Clustering of coassembly viruses together with similarly detected multiassembly viruses resulted in 25,355 species-level virus taxonomic units (vOTUs); 85.6% of these were unique to the coassembly, 12.3% were unique to the multiassembly, and the remaining 2.1% were shared. When clustering at the genus or family levels, the proportion of vOTUs unique to the coassembly remained roughly the same, while the proportion of vOTUs unique to the multiassembly shrank (4.2% genus level and 3.2% family level). These results indicate that, while multiassembly still captures some potentially valuable species-level metagenomic diversity, coassembly captures substantially more viral contigs from a broader phylogenetic distribution. Moreover, the read coverage of viruses unique to the coassembly was substantially lower than in those viruses found in both the coassembly and multiassembly, indicating, as with microbial MAGs, that the GRE coassembly may contain potentially rare, low-abundance viruses.

### Sequencing coverage and diversity.

To assess how thoroughly 3.4 Tbp of sequencing covers the LEF microbial communities, we applied Nonpareil (39) to the 95 GRE metagenomes. The fraction of a metagenomic community covered by a sequencing effort may bias the results due to insufficient coverage of less abundant microbes, resulting in fragmented, incomplete assemblies; thus, an assessment of the coverage of a sequencing effort given diversity in the community is useful. Nonpareil samples reads and analyzes the redundancy of k-mers to estimate coverage and predict the amount of sequencing effort required to achieve >95% of the diversity in an environment. The median diversity index, *N_d_*, computed by Nonpareil for the GRE data sets was 22.8, consistent with soil samples analyzed in reference 39. As expected, the bulk samples, which are not biased by stable isotope probing (SIP) fractionating, have a higher *N_d_* (median, 23.1) than the SIP fractions (median, 22.7). Nonpareil’s estimate of the projected sequencing to cover 95% of the sequence diversity (median of 1.25 Tbp based on the bulk samples) is exceeded by the 3.4 Tbp we have from the GRE. Allowing for bias that may have been introduced by the redox and SIP experiments, the GRE metagenomes may cover the majority of the microbial diversity in the LEF.

The implication that 3.4 Tbp of sequencing is possibly enough to cover 95% of the diversity in LEF soils is tempered by our finding that 87.5% of the reads mapped to contigs, leaving 12.5% of the reads unaccounted for. The JGI assembly release process excludes all scaffolds of <500 bp, and it is possible that a substantial fraction of the unmapped reads would map to these excluded contigs. It could also be that some rare biosphere microbes in the LEF are at such low abundance that 3.4 Tbp of sequencing still does not provide sufficient coverage to assemble them.

### Conclusions.

In summary, we present the coassembly, MAGs, and functional annotations of 3.4 Tbp of tropical soil metagenome data. We suggest that terabase-scale metagenome coassembly captures substantially more high-quality MAGs from a broader array of phyla than multiassembly of the same samples and is a useful tool for characterizing an environment’s full taxonomic diversity, including eukaryotic organisms and rare, low-abundance microbes and viruses. Multiassembly remains a valuable approach as, in addition to its computational feasibility, it may recover more species- and strain-level variation than coassembly. Thus, coassembly and multiassembly are complementary strategies for metagenome analysis, and we suggest that the optimal results can be achieved when both can be performed. As sequencing of environments goes deeper and bioinformatics methods improve, we may enter the era of the reference metagenome, in which high-quality MAGs may be assembled for the majority of a microbial community, and provide broad utility for the interpretation of experiments such as the GRE.

## MATERIALS AND METHODS

### Sample collection.

Several kilos of soil was collected from the Luquillo Experimental Forest soil, homogenized, and incubated as described in reference 15. Briefly, we collected surface soil from a mid-ridge position in the LEF near the El Verde Field Station and then conducted a well-replicated resolution redox oscillation study in the laboratory at Lawrence Livermore National Laboratory (LLNL), the Great Redox Experiment (GRE). These soils are pH 5 clay-rich volcanoclastic oxisols overlain by a tabunuco forest and receive 2,000 to 6,000 mm in annual rainfall. In 178 microcosms, soils were amended with ^13^C-labeled or unlabeled (^12^C) plant litter and incubated for 44 days under four treatments: (i) static anoxic, (ii) static oxic, (iii) flux 4-day (4 days anoxic, 4 days oxic), and (iv) flux 8-day (4 days anoxic, 8 days oxic). DNA was extracted from replicate microcosms harvested at 44 days using a modified Griffith’s protocol (40). DNA was then density fractionated on the LLNL HT-SIP pipeline (41). A total of 10 unfractionated bulk samples (2 replicates from each redox treatment and 2 time-zero original samples) and 86 stable isotope probing density fractions (2 samples per treatment/isotope, and the 5 to 6 heaviest fractions per sample) were submitted to the JGI for metagenomic sequencing. One sample failed during library prep and was abandoned.

### Genome sequencing.

Libraries were generated using either using the Kapa Biosystems library preparation kit (Roche) or the Nextera XT kit (Illumina), depending on available mass. For the Kapa Biosystems libraries, 200 ng of DNA was sheared to approximately 500 bp using an LE220 focused-ultrasonicator (Covaris). The sheared DNA fragments were size selected by double-solid phase reversible immobilization (SPRI), and then the selected fragments were end-repaired, A-tailed, and ligated with Illumina-compatible sequencing adaptors from IDT containing a unique molecular index barcode for each sample library. For the Nextera XT libraries, 2 ng of DNA was fragmented and adapter ligated. The ligated DNA fragments were enriched with 9 to 12 cycles of PCR and purified using SPRI beads (Beckman Coulter or Omega Bio-tek). Quantitative PCR (qPCR) was used to determine the concentration of the libraries using a LightCycler 480 real-time PCR instrument (Roche). Sequencing of the flow cell was performed on the NovaSeq (Illumina) sequencer using NovaSeq XP V1 reagent kits, S4 flow cell, following a 2 × 151-indexed run recipe.

### Metagenome assembly.

For the individual metaSPAdes assemblies, paired-end Illumina reads were trimmed and screened according to the documentation for BBTools (42) filtered reads and were read corrected using BFC (43) version r181 (bfc -1 -s 10g -k 21 -t 10). Reads with no mate pair were removed. The resulting reads were then assembled using metaSPAdes version 3.12.0 (12) using a range of kmers (spades.py -m 2000 –only-assembler -k 33,55,77,99,127 –meta -t 32 -1 -2). The entire filtered read set was mapped to the final assembly, and coverage information was generated using BBMap version 38.22 using default parameters except for “ambiguous=random.”

To generate the coassembly, 95 FASTQ files totaling 7.74 TB of filtered reads, representing 3.40 Tbp of sequence and 265 billion unique 31-mers, were coassembled with MetaHipMer2 (11) version 2.0.1.v2.0.0-110-gb87d7c1-Issue69 (mhm2.py -v –post-asm-align –post-asm-abd –checkpoint=yes –checkpoint-merged=no –pin=none –ranks-per-gpu = 7) on 512 nodes on the Oak Ridge National Laboratory (ORNL) Summit supercomputer, taking approximately 1 h 24 m. Following assembly, contigs smaller than 500 bp were removed. Alignment coverage information was computed internally in MetaHipMer2 using a method similar to merAligner (44) during postassembly processing. Alignments for binning and annotation purposes were computed with BBTools (42) version 38.90 (bbmap.sh nodisk=true interleaved=true ambiguous=random mappedonly=t trimreaddescriptions=t usemodulo=t fast=t). Coverage information was determined using BBTools version 38.79 (pileup.sh) and default parameters on the combined read set, and again for each of the 95 individual read sets. Bin- or contig-wise coverage information reported in this paper was taken from the Avg_fold field output by pileup.sh.

### Annotation.

Feature prediction and functional annotation of the assembled contigs were performed with version 5.0.24 of the IMG Annotation Pipeline (available for data set submission at https://img.jgi.doe.gov/submit). CRISPR elements were predicted via an in-house modified version of CRT-CLI version 1.2 (45) using the following search criteria: an element needs to have at least three repeats, search window size is set to 7 bp, minimum and maximum spacer length is set to 20 and 60 bp, respectively, and minimum and maximum repeat lengths are set to 20 and 50 bp, respectively. rRNA genes (5S, 16S, 23S), RNA regulatory features, and noncoding RNA genes were identified by comparing the contigs against the Rfam version 13.0 database (46) via cmsearch from the Infernal version 1.1.3 package (47) using the trusted cutoffs parameter (–cut_tc). Prediction of tRNAs was performed using the “bacterial” and “archaeal” search modes (-B/-A) of tRNAscan-SE version 2.0.8 (48). A combination of GeneMarkS-2 version 1.05 (49) (–Meta mgm_11.mod –incomplete_at_gaps 30) and Prodigal version 2.6.3 (50) (-p meta -m) was used to predict protein-coding genes.

Thereafter, the protein-coding genes were associated with functional annotations. KEGG Orthology (KO) terms and Enzyme Commission (EC) numbers were derived by running lastal 1066 from the LAST package (51) with default parameters against a reference database of isolate proteomes (IMG-NR 20190607). Comparison of the protein sequences against the remaining databases was performed with the HMMER version 3.1b2 (52) package, and specifically, via a thread-optimized version of hmmsearch (53). Assignments to version 15.0 of the TIGRFAM database (54), the frozen set of version 4.2.0 of the CATH-FunFam database (55), version 1.75 of the SUPERFAMILY database (56), version 01_06_2016 of the SMART database (57), and the updated 2014 COG models (58) were derived using a per-domain E value cutoff of 0.01 (–domE 0.01), whereas for Pfam-A assignments, the proteins were compared to version 30 of the Pfam database (59) using model-specific trusted cutoffs (–cut_tc).

Based on the name of their associated protein family, protein product names were then assigned in the order of priority: KO term > TIGRFAM > COG > Pfam. If none of the mentioned annotations were assigned, proteins were annotated as “hypothetical protein.”

Overlap resolution, filtering, and postprocessing of the various steps were executed as detailed in reference 19.

### Binning and MAG extraction.

MetaBAT version 2:2.15 (60) was used to generate the depths from the per-sample alignment files (jgi_summarize_bam_contig_depths –outputDepth) and generate the bins (metabat2 -m 3000 –minS 80 –maxEdges 500 –seed 1000), completeness and contamination were assessed with CheckM version 1.1.3 (checkm lineage_wf -x fa) (61), and taxonomy was assigned using GTDB-Tk version 1.3.0 and GTDB database release 95 (gtdbtk classify_wf –extension fa) (62). Bins were assigned as high, medium, or low quality based on the MiMAG standards (20). The multiassembly MAGs were generated by applying the same procedure to each of the individual added metaSPAdes assemblies, using each assembly’s read alignment file to generate the depths for MetaBAT. The 294 medium- and high-quality multiassembly MAGs are provided in Table S2.

### Construction of a concatenated marker gene phylogeny.

A three-subunit RNA polymerase (COG00085, COG00086, and COG0202) concatenated marker gene tree was constructed by combining a set of reference genomes spanning the bacterial and archaeal domains together with the GRE query genomes, i.e., the GRE coassembly and multiassembly MAGs. The set of reference genomes was collected by clustering the full set of public Integrated Microbial Genomes (IMG) isolate genomes using Cd-hit (63) version 4.8.1 to cluster the RNA polymerase gene (rpoB) at 80%, producing a set of references that was roughly unique at the genus to family taxonomic level. The GRE MAGs were dereplicated by grouping MAGs into species-level groups using a Mash (22) version 2.0 cutoff distance of 0.05, followed by clustering with mcl (64) version 14-137 with an inflation parameter of 1.5. The dereplicated set of MAGs and the reduced set of archaeal and bacterial reference genomes were passed through the SGTree version 0.0.10 pipeline (65). Briefly, this pipeline extracts the set of three marker genes from the set of query and reference genomes using hmmsearch version 3.1b2 (52), performs alignments of each marker with MAFFT (66) version v7.490 (2021/Oct/30) using the mafft-linsi option, trims alignments with trimAl version 1.4 (67), and removes sites when more than 90% of taxa contain a gap. The presence of all 3 subunits was required for a reference and/or query genome to be included into the tree; thus, due to various levels of MAG completeness, not all MAGs are included in Fig. 1. Finally, individual protein alignments were concatenated, followed by maximum likelihood tree construction with IQ-TREE (68) multicore version 1.6.1 using the WAG substitution model with 1,000 bootstraps. Trees were visualized with ggtree (69) version ggtree_3.2.1, and Faith’s phylogenetic diversity (PD, the sum of all branch lengths separating taxa in a community) was computed using the R Picante package (70) version picante_1.8.2. Phylum-level taxonomic designations were assigned using GTDB-Tk (62).

### Rare biosphere microbes.

Taxonomic assignment of low-quality MAGs was performed with GTDB-Tk version 1.5.1 (gtdbtk classify_wf –extension fa) (62). Completeness and contamination metrics were calculated with CheckM during the binning procedure detailed above. Bin contig coverage information was calculated with BBTools as described above in the metagenome assembly details. Coassembly and multiassembly coverage of rare biosphere bins as a whole were calculated as contig length-weighted means of Avg_fold values for each contig.

### Eukaryotic MAGs.

To detect possible eukaryotic MAGs, EukCC version 2.1.2 (32) (eukcc folder –threads 32 –suffix fa) was run on the nucleotide sequences of the low-quality bins (completion, <50%; contamination, <10%) using the database downloaded from http://ftp.ebi.ac.uk/pub/databases/metagenomics/eukcc/.

The fungal MAG’s completeness was estimated using BUSCO version 5.4.3 (36) (busco -m genome -c 32 -q -f) and the fungi_odb10 database and was inferred to be in the *Coniochaeta* clade based on BLAST searches against the MycoCosm (71) and NCBI nonredundant (nr) (72) databases.

### Virus identification and clustering.

To identify and taxonomically classify viral genomes in the multiassembly and coassembly, contigs with at least 3,000 bp were processed with geNomad version 1.1.0 (available at https://github.com/apcamargo/genomad) (38). Composition-based score calibration was employed to set the false-discovery rate to 5% (–enable-score-calibration –max-fdr 0.05), and the presence of at least one virus hallmark was required (–min-virus-hallmarks 1). Genome completeness was estimated using CheckV version 1.0.1 (database version 1.4) (73).

To cluster the viral genomes identified across all the assemblies into viral taxonomic units (vOTUs), we first performed an all-versus-all BLAST (74) search (version 2.13.0+, parameters: -task megablast -evalue 1e-5 -max_target_seqs 20000) to align pairs of similar sequences. Next, the average nucleotide identity (ANI) and the aligned fraction (AF) of each pair were computed from the alignments, and a graph was constructed by connecting viral contigs with an ANI of ≥95% and AF of ≥85% (75). Last, sequences were clustered into vOTUs using the Leiden algorithm (76) (from the igraph Python library, version 0.9.10, resolution parameter = 1.0). The code used for vOTU-level clustering can be found at https://github.com/apcamargo/bioinformatics-snakemake-pipelines/tree/main/genome-ani-leiden-clustering-pipeline. Genomes were also clustered at the genus and family levels using pairwise average amino acid identities (AAI) computed from the output of an all-versus-all DIAMOND (version 2.0.15.153) search, as described previously (77). Scripts used for AAI-based clustering can be found at https://github.com/snayfach/MGV/tree/master/aai_cluster.

### Metagenome coverage and diversity.

To estimate metagenome coverage and diversity, we used Nonpareil3 version 3.3.03 (39) (nonpareil -T kmer -f fastq -t 32 -R 110000). FASTQ files were pooled into 23 groups according to the SIP/bulk experiment (Table S3) and split by read pair using BBTools version 38.75 (reformat.sh int=t). Because Nonpareil analyzes only one member of each paired read, and because ours is a paired-read data set, we take the computed LRstar of 1.25 Tbp to obtain our estimate of how much sequencing would be required to cover 95% of the diversity in the LEF.

### Data availability.

The GRE coassembly, high- and medium-quality MAGs, and annotations are available in the IMG/MER Database under the taxon object ID 3300047160. The corresponding data for the 95 individual metagenomes that were individually assembled for this work can be found by following the related sample links from the microbiome details page. The metadata for these samples can be found in the GOLD database (https://gold.jgi.doe.gov/) under the analysis project identifier Ga0500728. Raw reads can be found in the NCBI SRA database using the accession numbers provided in Table S4.
